# Prevalence, determinants and association of unawareness of diabetes, hypertension and hypercholesterolemia with poor disease control in a multi-ethnic Asian population without cardiovascular disease

**DOI:** 10.1186/s12963-019-0197-5

**Published:** 2019-12-05

**Authors:** Ryan E. K. Man, Alvin Hong Wei Gan, Eva K. Fenwick, Alfred Tau Liang Gan, Preeti Gupta, Charumathi Sabanayagam, Nicholas Tan, Kah Hie Wong, Tien Yin Wong, Ching-Yu Cheng, Ecosse L. Lamoureux

**Affiliations:** 1 Singapore Eye Research Institute, Singapore National Eye Centre, 20 College Rd, The Academia, Discovery Tower Level 6, Singapore, 169856 Singapore; 2 Duke-NUS Medical School, Singapore, Singapore; 3 Singapore National Eye Centre, Singapore, Singapore; 4 National University of Singapore, Singapore, Singapore

**Keywords:** Awareness, Cardiovascular risk factor, Hypercholesterolemia, Hypertension, Diabetes, Determinant

## Abstract

**Background:**

To explore the prevalence and determinants of unawareness of diabetes, hypertension and hypercholesterolemia and its association with poor disease control in a multi-ethnic Asian population without cardiovascular disease (CVD).

**Methods:**

We included 6904 Chinese, Malay and Indian individuals (mean age [SD] 58.2 [10.2] years; 52.6% female) with diabetes, hypertension and/or hypercholesterolemia from the cross-sectional population-based Singapore Epidemiology of Eye Diseases study (2004–2011). Diabetes was defined as random blood glucose ≥ 11.1 mmol/L or HbA1c > 6.5% or self-reported use of diabetes medication; hypertension as systolic blood pressure ≥ 140 mmHg or diastolic blood pressure ≥ 90 mmHg or self-reported use of anti-hypertensive treatment; and hypercholesterolemia as total cholesterol ≥ 6.2 mmol/L or self-reported use of lipid-lowering medications. Unawareness was based on participants’ answers to the questions: “Did your medical practitioner ever tell you that you have diabetes/hypertension/high cholesterol?” The determinants of unawareness, and its association with poor disease control, were assessed using multivariable binary logistic regression models adjusted for known potential confounders.

**Results:**

Of the 2380 (34.5%), 5386 (78.0%) and 3607 (52.2%) with diabetes, hypertension and hypercholesterolemia, respectively, unawareness rates were 30.7%, 43.1% and 40.9%, respectively. Having a higher BMI, particularly if obese, and Malay ethnicity were associated with greater unawareness of diabetes; Malay and Indian ethnicities and current smoking with greater unawareness of hypertension; and education ≤6 years, current smoking, and blue collar jobs or unemployment with greater unawareness of hypercholesterolemia (all *P* < 0.05). Lack of awareness of each condition was independently associated with poorer disease control in the case of hypertension and hypercholesterolemia, while the converse was true for diabetes (all *P* < 0.05).

**Conclusions:**

Unawareness of diabetes, hypertension, or hypercholesterolemia is high in Singapore, with risk factors varying across all three diseases, although Malay ethnicity is a consistent one. Unawareness was also associated with poor management for hypertension and hypercholesterolemia. Public health education and screening programs should target at-risk individuals, especially Malays, to reduce the likelihood of incident CVD.

## Background

An individual’s awareness of the presence of major risk factors of cardiovascular disease (CVD), i.e. diabetes, hypertension and hypercholesterolemia, plays an important role its prevention [1–3], as it will more likely lead to treatment seeking and disease self-management [4, 5]. A substantial proportion of the general population who suffer these conditions, however, are unaware that they have these CVD risk factors [1, 6]. For instance, studies in Asia have demonstrated unawareness rates of between 13.2% [2] and 30.8% [1, 6] for diabetes and hypertension, respectively.

Understanding the extent of unawareness of these three conditions in individuals without a history of CVD, together with their associated determinants, is important as it allows researchers and policy planners to implement informed public health interventions and educational programs and hence reduce their risks of developing CVD. However, most studies exploring unawareness of these three main CVD risk factors have been conducted in Western populations [3, 7–10], with comparatively limited information available for Asian countries. This is unfortunate as the differences in culture, health care systems and access to care between Western and Asian societies may also mean differing rates of unawareness and associated determinants of these CVD risk factors. Therefore, addressing this relative paucity of data is critical as there are approximately 4.4 billion individuals living in Asia, comprising almost 60% of the global population. Moreover, the populations that current research in Asia has drawn upon are relatively homogenized, e.g. in the very elderly [6] or in specific races, such as Indians [1], which limits generalizability of the results. Clarity is also lacking around potential ethnic differences in the unawareness rates of these three conditions amongst the three major ethnic groups in Asia, i.e. the Chinese, Malays and Indians. Singapore offers a unique opportunity to examine this gap in knowledge as it is an urbanized city state comprising the three main Asian ethnic groups, with all three ethnicities having access to the same information dissemination and healthcare systems.

We therefore assessed the individual prevalence and associated determinants of unawareness of diabetes, hypertension and hypercholesterolemia in a well-characterized multi-ethnic adult Singaporeans without CVD. We hypothesize that unawareness of these three diseases in this urbanized cityscape will be relatively low, although unawareness of an individual’s own condition will be associated with poorer disease control.

## Methods

### Study population

The Singapore Epidemiology of Eye Diseases Study is a population-based cross-sectional study of 3353 Chinese, 3280 Malays and 3400 Indians aged 40–80 years, conducted in Singapore from 2004 to 2011. The study methodology has been published elsewhere [11, 12]. All participants were made aware of the study protocol, and written informed consent was obtained. The study adhered to the Declaration of Helsinki, with ethics approval obtained from the Singapore Eye Research Institute (SERI) Institutional Review Board.

For the purpose of this study, we excluded 1116 individuals with self-reported CVD (comprising angina, stroke and heart attack), as well as those not suffering from any of the three conditions under investigation, i.e. diabetes, hypertension and hypercholesterolemia (*N* = 2013), leaving a total of 6904 participants with any one of the conditions, alone or in combination, for analysis.

### Definition of outcomes

#### Diabetes

Random glucose and HbA1c were measured from non-fasting blood samples at the National University Hospital Reference Laboratory which is accredited by the College of American Pathologists. HbA1c assay was carried out using high-performance liquid chromatography cation exchange chromatography system implemented on a Biorad variant II analyzer. The assay was accredited by the National Glycoprotein Standardization Program with controls traceable to the Diabetes Control and Complications Trial [13].

Individuals were classified as having diabetes if they had a random blood glucose ≥ 11.1 mmol/L and/or HbA1c > 6.5% or self-reported the use of diabetes medication. All medication use was cross-checked with actual medicine labels where possible to lessen the chances of recall bias or interviewer error.

#### Hypertension

Systolic and diastolic blood pressures (SBP and DBP, respectively) were measured by trained clinic research assistants using a regularly calibrated digital automatic blood pressure monitor (Dinamap model Pro Series DP110X-RW, 100 V2; GE Medical Systems Information Technologies, Inc., Milwaukee, WI), after the subject was seated for at least 5 min. BP was measured twice, 5 min apart. A third measurement was made if SBP differed by more than 10 mmHg or DBP by more than 5 mmHg. The average between the two closest readings was then utilized in analyses. Individuals were defined as having hypertension if they had an SBP ≥ 140 mmHg or DBP ≥ 90 mmHg or were on self-reported use of anti-hypertensive treatment. Per the use of diabetes medications, all anti-hypertensive medication use was cross-checked with actual medicine labels where possible to lessen the chances of recall bias or interviewer error.

#### Hypercholesterolemia

Forty millilitres of venous blood was collected to measure serum lipids (total, low-density lipoprotein, and high-density lipoprotein cholesterol). All serum biochemistry tests were carried out at the National University Hospital Reference Laboratory. Individuals were classified as having hypercholesterolemia if they had a total cholesterol ≥ 6.2 mmol/L or self-reported the use of lipid-lowering medications. Similarly, all lipid-lowering medication use was cross-checked with actual medicine labels where possible to lessen the chances of recall bias or interviewer error.

### Unawareness

Unawareness of diabetes, hypertension and hypercholesterolemia was determined if the individual indicated “no” to the following question(s), “Did your medical practitioner ever tell you that you have diabetes? hypertension? or high cholesterol?”. Responses other than “yes” or “no” were considered missing and excluded from these analyses.

### Assessment of covariates

Participants underwent a comprehensive, standardized examination at SERI to collect clinical and questionnaire data [12, 14]. Demographic information, sociodemographic characteristics (education, income level, occupation, marital status, housing type), lifestyle factors (smoking, alcohol, diet), and self-reported family and medical history (diabetes, hypertension, high cholesterol, thyroid diseases, stroke, cardiovascular disease, current medication use) were collected via questionnaires administered by trained interviewers in English, Malay, Chinese and Tamil according to participant preference. Height and weight were measured using a wall-mounted adjustable measuring scale and a calibrated scientific weight scale, respectively. BMI (body mass index) was calculated as weight (kg) divided by height in meters squared (kg/m^2^). BMI was further categorized into underweight/normal weight (< 23 kg/m^2^), overweight (23–27.5 kg/m^2^) and obese (> 27.5 kg/m^2^) based on the Asian BMI cut-points recommended by the World Health Organization [15].

### Statistical analyses

Analyses were performed using Stata version 14.2 (StataCorp LP, College Station, TX, USA). Within each disease-positive sample, we estimated the crude prevalence of unawareness of that disease, overall and stratified by 10-year age groups and ethnicity. To evaluate the determinants of unawareness of the three diseases, we also compared background, sociodemographic, and clinical and biochemical characteristics between patients who were aware and unaware of having each disease using the chi-squared test or Fisher’s exact test for categorical variables as appropriate, and the student’s *t* test for continuous variables. Variables that were significantly associated with the outcomes in univariable analyses (i.e. *P* < 0.05; see Table 1) or those that were found to be associated with unawareness of the condition(s) in previous studies were then assessed for an independent association with unawareness of each disease by simultaneous inclusion as covariables in a multivariable logistic regression model. These variables include age, gender, race, BMI (continuously and categorically), income, occupation, marital status, housing, number of non-CVD-related comorbidity, and mutually for each other.

**Table 1:** Baseline characteristics of patients by lack of awareness of diabetes, hypertension and hypercholesterolemia

	Diabetes			Hypertension			Hypercholesterolemia		
	Mean (SD)/*N* (%)			Mean (SD)/*N* (%)			Mean (SD)/*N* (%)		
Variable	Aware (*N* = 1649)	Unaware (*N* = 731)	*P* value	Aware (*N* = 3067)	Unaware (*N* = 2319)	*P* value	Aware (*N* = 2132)	Unaware (*N* = 1475)	*P* value
Age (years)	62.0 (9.59)	59.2 (10.4)	*< 0.001*	62.8 (9.67)	59.7 (10.4)	*< 0.001*	61.6 (9.52)	59.0 (10.2)	*< 0.001*
Body mass index (kg/m^2^)	26.7 (4.58)	27.3 (4.98)	*0.001*	26.5 (4.81)	25.3 (4.54)	*< 0.001*	26.0 (4.42)	25.3 (4.54)	*< 0.001*
Number of self-reported comorbid conditions*	0.61 (0.75)	0.39 (0.61)	*< 0.001*	0.54 (0.72)	0.40 (0.62)	*< 0.001*	0.56 (0.74)	0.37 (0.59)	*< 0.001*
Self-reported hypercholesterolemia	998 (61.9)	216 (30.4)	*< 0.001*	1701 (57.0)	424 (18.6)	*< 0.001*	–	–	–
Self-reported hypertension	1008 (61.2)	270 (37.1)	*< 0.001*	–	–	–	1337 (62.9)	328 (22.3)	*< 0.001*
Self-reported diabetes	–	–	–	1008 (33.0)	329 (14.2)	*< 0.001*	770 (36.2)	180 (12.2)	*< 0.001*
Gender									
Male	759 (46.0)	376 (51.4)	*0.015*	1354 (44.1)	1180 (50.9)	*< 0.001*	928 (43.5)	660 (44.7)	0.5
Female	890 (54.0)	355 (48.6)		1713 (55.9)	1139 (49.1)		1204 (56.5)	815 (55.3)	
Race									
Malay	519 (31.5)	337 (46.1)	*< 0.001*	1022 (33.3)	931 (40.1)	*< 0.001*	470 (22.0)	602 (40.8)	*< 0.001*
Indian	771 (46.8)	249 (34.1)		968 (31.6)	641 (27.6)		796 (37.3)	393 (26.6)	
Chinese	359 (21.8)	145 (19.8)		1077 (35.1)	747 (32.2)		866 (40.6)	480 (32.5)	
Income *<* S$1000	1059 (65.2)	406 (56.7)	*< 0.001*	1956 (65.0)	1328 (58.2)	*< 0.001*	1229 (58.8)	843 (57.9)	0.6
≤ 6 years education	1139 (69.1)	494 (67.6)	0.5	2117 (69.1)	1520 (65.6)	*0.007*	1308 (61.4)	960 (65.1)	*0.022*
Current smoker	190 (11.5)	129 (17.6)	*< 0.001*	311 (10.2)	402 (17.4)	*< 0.001*	214 (10.1)	275 (18.7)	*< 0.001*
Alcohol drinker	117 (7.12)	46 (6.29)	0.5	221 (7.22)	214 (9.24)	*0.007*	168 (7.88)	98 (6.66)	0.2
Lives alone	94 (5.71)	34 (4.66)	0.3	165 (5.40)	107 (4.62)	0.2	107 (5.03)	80 (5.44)	0.6
Works outdoor	92 (5.58)	46 (6.29)	0.5	154 (5.02)	153 (6.60)	*0.014*	101 (4.74)	102 (6.92)	*0.005*
Occupation									
Professionals and office workers	224 (13.6)	129 (17.6)	*< 0.001*	430 (14.0)	376 (16.2)	*< 0.001*	411 (19.3)	227 (15.4)	*< 0.001*
Service workers, production workers or cleaners	432 (26.2)	241 (33.0)		808 (26.4)	738 (31.8)		568 (26.7)	491 (33.3)	
Homemakers, retirees, unemployed or others	992 (60.2)	361 (49.4)		1827 (59.6)	1205 (52.0)		1152 (54.1)	757 (51.3)	
Marital Status									
Never married	63 (3.83)	35 (4.79)	*0.003*	142 (4.63)	128 (5.53)	*< 0.001*	99 (4.65)	102 (6.92)	*0.006*
Married	1225 (74.4)	580 (79.5)		2251 (73.4)	1784 (77.1)		1638 (76.9)	1090 (74.0)	
Separated or divorced	53 (3.22)	24 (3.29)		102 (3.33)	94 (4.06)		72 (3.38)	67 (4.55)	
Widowed	306 (18.6)	91 (12.5)		570 (18.6)	309 (13.3)		322 (15.1)	214 (14.5)	
Housing									
5 room HDB and above	426 (25.8)	196 (26.8)	0.6	863 (28.2)	621 (26.8)	0.3	712 (33.4)	384 (26.1)	*< 0.001*
3-4 room HDB or less	1222 (74.2)	535 (73.2)		2200 (71.8)	1697 (73.2)		1418 (66.6)	1090 (73.9)	

*HDB* Housing Development Board

*Includes ocular (cataract, myopia, age-related macular degeneration, glaucoma, diabetic retinopathy, eye trauma) and systemic non-CVD (e.g. thyroid disease) conditions

Values in italics: *P* <0.05

To estimate the independent relationship between disease unawareness and clinical control, we constructed a multivariable logistic regression model with unawareness as the exposure and poor clinical control of the disease as the outcome (i.e. SBP ≥ 140 mmHg or DBP ≥ 90 mmHg for hypertension, HbA1c > 7% for diabetes and total cholesterol ≥ 6.2 mmol/L for hypercholesterolemia), adjusted for variables that were associated with the outcomes in univariable analyses (see Additional file 1: Table S1) or were found to be associated with the respective poor disease control in previous studies. These variables include age, gender, ethnicity, body mass index, self-reported non-cardiovascular comorbidity, income, education, smoking, occupation, marital status, housing, duration of diabetes (for lack of awareness of diabetes only), and mutually for each other. All estimates were presented with 95% confidence intervals.

## Results

There were 2380 (34.5%) individuals with diabetes (mean age [SD] 61.1 [9.9] years; 52.3% female), 5386 (78.0%) with hypertension (mean age [SD] 61.5 [10.1] years; 53.0% female) and 3607 (52.2%) with hypercholesterolemia (mean age [SD] 60.5 [9.9] years; 56.0% female; Table 1). The rates of unawareness were 30.7% (95% CI [confidence intervals] 28.9%, 32.6%), 43.1% (41.7%, 44.4%) and 40.9% (39.3%, 42.5%), respectively (Table 2). Age-stratified analyses revealed that unawareness was highest amongst those aged 40–49 years and lowest in those aged over 70 years for all three conditions (*P* < 0.001; Table 2). In addition, amongst the three main ethnicities, unawareness was highest in Malays for all three conditions (*P* < 0.001; Table 3).

**Table 2:** Prevalence of the lack of disease awareness in those with diabetes, hypertension and hypercholesterolemia by age group

	Overall		40-49 years		50-59 years		60-69 years		≥ 70 years		*P*^†^
Disease	*N*^‡^	Prevalence (%)	*N*^‡^	Prevalence (%)	*N*^‡^	Prevalence (%)	*N*^‡^	Prevalence (%)	*N*^‡^	Prevalence (%)	
Diabetes	2380	30.7 (28.9, 32.6)	376	45.2 (40.3, 50.3)	713	31.7 (28.4, 35.2)	764	25.7 (22.7, 28.9)	527	26.4 (22.8, 30.3)	*<* 0.001
Hypertension	5386	43.1 (41.7, 44.4)	845	60.0 (56.7, 63.3)	1562	46.2 (43.8, 48.7)	1689	37.1 (34.8, 39.4)	1290	36.0 (33.4, 38.6)	*<* 0.001
Hypercholesterolemia	3607	40.9 (39.3, 42.5)	614	55.0 (51.1, 58.9)	1151	42.2 (39.4, 45.1)	1103	33.9 (31.2, 36.8)	739	37.5 (34.1, 41.0)	*<* 0.001

Excluding those with CVD

^‡^Number with disease and who provided valid responses (yes/no) to questions on disease status awareness

^†^Chi-squared test comparing proportions across age groups

**Table 3:** Prevalence of the lack of disease awareness in those with diabetes, hypertension and hypercholesterolemia by ethnicity

	Chinese		Malay		Indian		
Disease	*N*^‡^	Prevalence (%)	*N*^‡^	Prevalence (%)	*N*^‡^	Prevalence (%)	*P* value^†^
Diabetes	504	28.8 (25.0 to 32.9)	856	39.4 (36.2 to 42.7)	1020	24.4 (21.9 to 27.1)	*<* 0.001
Hypertension	1824	41.0 (38.7 to 43.2)	1953	47.7 (45.5 to 49.9)	1609	39.8 (37.5 to 42.3)	*<* 0.001
Hypercholesterolemia	1346	35.7 (33.1 to 38.3)	1072	56.2 (53.2 to 59.1)	1189	33.1 (30.4 to 35.8)	*<* 0.001

Excluding those with CVD

^‡^Number with disease and who provided valid responses (yes/no) to questions on disease status awareness

^†^Chi-squared test comparing proportions across ethnicities

In multivariable models, we found that Malay ethnicity (compared to Chinese) was associated with higher likelihood of subjects being unaware of three conditions individually (all *P* < 0.05; Table 4). Current smokers (compared to non/ex-smokers) were more likely to be unaware of having hypertension and hypercholesterolemia (*P* < 0.05 for both; Table 4). Other determinants of non-awareness were higher BMI for diabetes, particularly individuals who were obese; Indian ethnicity for hypertension; and ≤ 6 years of education and unemployment and working blue collar jobs (compared to white collar professionals) for hypercholesterolemia (*P* < 0.05 for all, Table 4). In contrast, self-reporting the concomitant presence of any of the three conditions was associated with greater likelihood of awareness of the three CVD risk factors. Similarly, higher self-reported number of non-CVD related comorbidities was associated with greater awareness of diabetes and hypercholesterolemia; and higher BMI (both overweight and obese) with greater awareness of hypertension and hypercholesterolemia, Other determinants of awareness include Indian ethnicity (compared to Chinese) and unemployment (compared to working in white collar professions) for diabetes, older age for hypertension, and marriage for hypercholesterolemia (all *P* < 0.05; Table 4).

**Table 4:** Determinants of lack of awareness of diabetes, hypertension and hypercholesterolemia in multivariable models

	Diabetes		Hypertension		Hypercholesterolemia	
Variable	OR (95% CI)	*P* value	OR (95% CI)	*P* value	OR (95% CI)	*P* value
Age (years)	1.00 (0.99, 1.01)	0.9	0.97 (0.96, 0.98)	*< 0.001*	0.99 (0.98,1.00)	0.05
40-49 years	Ref		Ref		Ref	
50-59 years	1.28 (0.95, 1.71)	*0.099*	0.72 (0.59, 0.87)	*0.001*	0.72 (0.57, 0.91)	*0.006*
60-69 years	1.24 (0.89, 1.71)	*0.2*	0.51 (0.41, 0.63)	*< 0.001*	0.67 (0.52, 0.87)	*0.003*
≥ 70 years	1.04 (0.70, 1.53)	*0.9*	0.43 (0.34, 0.56)	*< 0.001*	0.79 (0.57, 1.08)	*0.1*
Body mass index (kg/m^2^)	1.04 (1.02, 1.06)	*< 0.001*	0.94 (0.92, 0.95)	*< 0.001*	0.98 (0.96, 1.00)	*0.018*
Normal/underweight	Ref		Ref		Ref	
Overweight	1.02 (0.78, 1.35)	*0.880*	0.75 (0.65, 0.88)	*< 0.001*	0.79 (0.66, 0.96)	*0.017*
Obese	1.50 (1.13, 1.98)	*0.005*	0.52 (0.43, 0.61)	*< 0.001*	0.75 (0.60, 0.94)	*0.011*
Number of self-reported non-CVD comorbid conditions*	0.71 (0.60, 0.83)	*< 0.001*	0.93 (0.84, 1.02)	0.1	0.80 (0.70, 0.90)	*< 0.001*
Self-reported hypercholesterolemia	0.34 (0.27, 0.41)	*< 0.001*	0.22 (0.19, 0.25)	*< 0.001*	-	-
Self-reported hypertension	0.51 (0.41, 0.64)	*< 0.001*	–	–	0.20 (0.16, 0.23)	*< 0.001*
Self-reported diabetes	–	–	0.52 (0.44, 0.61)	*< 0.001*	0.32 (0.26, 0.40)	*< 0.001*
Gender						
Male	Ref		Ref		Ref	
Female	0.99 (0.77, 1.28)	0.9	0.89 (0.76, 1.04)	0.1	0.97 (0.80, 1.18)	0.8
Ethnicity						
Chinese	Ref		Ref		Ref	
Malay	1.33 (1.01, 1.75)	*0.046*	1.44 (1.23, 1.69)	*< 0.001*	2.68 (2.18, 3.31)	*< 0.001*
Indian	0.58 (0.44, 0.76)	*< 0.001*	1.24 (1.05,1.46)	*0.010*	1.11 (0.91, 1.35)	0.3
Income *<* S$1000	0.94 (0.71, 1.24)	0.7	0.93 (0.78, 1.12)	0.5	0.90 (0.72, 1.13)	0.4
≤ 6 years education	1.10 (0.86, 1.41)	0.4	1.00 (0.85, 1.17)	0.9	1.29 (1.05, 1.57)	*0.013*
Alcohol drinker	0.97 (0.64, 1.46)	0.9	1.01 (0.79, 1.28)	0.9	0.85 (0.62, 1.16)	0.3
Current smoker	1.17 (0.87, 1.58)	0.3	1.29 (1.06, 1.58)	*0.012*	1.35 (1.05, 1.74)	*0.018*
Occupation						
Professionals and office workers	Ref		Ref		Ref	
Service workers, production workers or cleaners	0.85 (0.62, 1.18)	0.3	1.02 (0.82, 1.26)	0.9	1.32 (1.02,1.69)	*0.033*
Homemakers, retirees, unemployed or others	0.65 (0.45, 0.93)	*0.017*	1.12 (0.89, 1.41)	0.3	1.54 (1.16, 2.05)	*0.003*
Marital Status						
Never married	Ref		Ref		Ref	
Married	0.90 (0.55, 1.48)	0.7	1.20 (0.90, 1.61)	0.2	0.58 (0.41, 0.81)	*0.002*
Separated or divorced	0.86 (0.42, 1.76)	0.7	1.23 (0.80, 1.89)	0.3	0.70 (0.42, 1.17)	0.2
Widowed	0.71 (0.40, 1.26)	0.2	1.22 (0.87, 1.72)	0.2	0.83 (0.55, 1.26)	0.4
Housing						
5 room HDB and above	Ref		Ref		Ref	
3–4 room HDB or less	0.80 (0.63, 1.02)	0.07	1.03 (0.88, 1.20)	0.7	1.13 (0.94, 1.37)	0.2

*HDB* Housing Development Board

Includes ocular (cataract, myopia, age-related macular degeneration, glaucoma, diabetic retinopathy, eye trauma) and systemic non-CVD (e.g. thyroid disease) conditions

Values in italics: *P* <0.05

Table 5 shows that unawareness of hypertension and hypercholesterolemia was independently associated with a 7- and 14-fold increased likelihood of poor disease control, respectively. Conversely, unawareness of having diabetes was associated with increased likelihood of having good glycaemic control after adjustment for potential confounders.

**Table 5:** Association between lack of awareness of diabetes, hypertension, and hypercholesterolemia and respective poor clinical control in multivariable models

	Diabetes^†^		Hypertension^‡^		Hypercholesterolemia^#^	
	OR (95% CI)	*P* value	OR (95% CI)	*P* value	OR (95% CI)	*P* value
Lack of awareness	0.51 (0.40, 0.66)	*< 0.001*	7.65 (6.18, 9.46)	*< 0.001*	14.58 (11.41, 18.63)	*< 0.001*

Adjusted for age, gender, ethnicity, body mass index, self-reported non-cardiovascular comorbidity, income, education, smoking, occupation, marital status, housing

^†^Additionally adjusted for duration of diabetes, systolic blood pressure, total cholesterol and HDL cholesterol, self-reported hypertension and self-reported hypercholesterolemia

^‡^Additionally adjusted for HbA1c, total cholesterol and HDL cholesterol, self-reported diabetes and self-reported hypercholesterolemia

^#^Additionally adjusted for HbA1c, systolic blood pressure, self-reported diabetes and self-reported hypertension

Values in italics: *P* <0.05

## Discussion

In this large population-based study of over 6900 multi-ethnic Asian adults, we showed that the prevalence of unawareness of hypertension (43.7%) was highest amongst the three CVD risk factors assessed, followed by hypercholesterolemia (40.9%) and diabetes (30.7%). Malay ethnicity was associated with a lack of awareness of all three conditions, followed by smoking (for hypertension and hypercholesterolemia). Importantly, self-reported awareness of concomitant CVD risk factors, or other non-CVD comorbidities, were strongly associated with greater awareness of having any of the three diseases under investigation. Our findings hence suggest that public education campaigns to increase awareness of CVD risk factors and promote the importance of annual health checks are needed to reduce high levels of unawareness of the three major CVD risk factors in adults aged ≥ 40 years in Singapore, particularly amongst Malay individuals and smokers.

Our finding that the unawareness rates of hypertension and hypercholesterolemia were higher than diabetes is corroborated by previous population-based studies [16–18]. For instance, Koreans ≥ 50 years reported hypercholesterolemia and hypertension unawareness rates of 53.3% and 27%, respectively, compared to diabetes which was significantly lower at 20.7% [16]. Our high diabetes awareness rates could partially be attributed to an extensive diabetes public health education and screening campaign in response to Singapore having the second highest rates of diabetes amongst developed nations [19]. Interestingly, an earlier study of the awareness of diabetes in Malay individuals by our group reported substantially lower lack of awareness (13.2%) [2] compared to the current study (30.7%). Two possible factors contributing to these differences include the fact that the previous report included individuals with self-reported CVD, which may have contributed to increased awareness, as well as the use of HbA1c to define diabetes in the current study, which more closely conforms with that recommended by the American Diabetes Association [20].

Previous studies on the unawareness rates of hypertension in Singaporean individuals have reported rates of 30.2% [6] and 48.2% [21], which differ from our finding (43.1%). Disparities could be due to differences in sample population and size, as well as areas surveyed. In addition, our finding that 40.9% of the population is unaware of having hypercholesterolemia is markedly higher than that reported previously in Singapore (17.3%) [22]. However, this difference may be due to the differing methods of disease classification, as Khoo and colleagues utilized LDL-cholesterol levels in their classification [22], while our study assessed total cholesterol levels instead. All in all, it is concerning that in spite of the relative ease of access to information and health care in Singapore, which is one of the most urbanized societies globally [23], over a third of patients with any of the three major CVD risk factors, alone or in combination, are still unaware of their condition(s). Our results therefore suggest that priority should be given towards developing public health strategies to raise awareness of these conditions to prevent the development of CVD and its associated morbidities.

Interestingly, when we looked more closely at the data, we found that there was a small proportion of individuals who professed a lack of awareness of their condition, in spite of reporting using medications to control the disease (Additional file 1: Table S2). This phenomenon appears to be common to Asian societies, where elderly individuals depend heavily on their caregivers to guide them in their activities of daily living. These individuals usually have little to no education and can only communicate in their native dialect, making day-to-day communication with others who do not speak the dialect difficult. As such, these individuals are often not apprised of their condition (since their poor grasp of medical jargon makes understanding difficult) and are simply told that they had to take the medication for their continued good health. We found within this sample of persons who were on medication, yet lacked awareness of their condition, that over 50% of them (73.5%, 63.6% and 59.6% for diabetes, hypertension and hypercholesterolemia, respectively) had primary and below education. Of these, over 65% were aged ≥ 60 years (80.0%, 65.7% and 78.9% for diabetes, hypertension and hypercholesterolemia, respectively). Hence, it is likely that their caregivers have indeed communicated with their doctors on the elderly participant’s behalf. Previous work has confirmed the presence of this phenomenon, e.g. Pirasath and colleagues found that ~ 14% of individuals attending a hypertensive clinic were unaware of having the disease [1]. Our results indicate a need for awareness campaigns to utilize simplified language and descriptions about these three CVD risk factors in order to improve awareness in this group of individuals.

Similar to other studies conducted in Singapore [6, 21], we observed that Malay individuals were more likely to be unaware of having diabetes, hypertension and hypercholesterolemia compared to Chinese and Indian individuals, independent of known factors predisposing towards lack of awareness such as low education and income levels [6, 16, 24]. Such consistent findings across different studies and diseases suggest that further research, both qualitative and quantitative, is needed to understand the reasons underlying this ethnic disparity in the lack of awareness of the three major CVD risk factors.

Our findings that lower BMI (both continuously and categorically), lower education levels, Indian ethnicity, smoking and unemployment/blue collar jobs were independently associated with lack of awareness of one or more of the three CVD risk factors is supported by data from other studies [21, 22]. That these factors are consistently being associated with lack of disease awareness is concerning as the implication is that public health education programs designed to raise awareness and promote screening for these diseases are not reaching at-risk individuals. In contrast, our observation that a higher BMI was associated with greater unawareness of diabetes is novel. It is particularly interesting that obesity was independently associated with greater unawareness of this disease as we would expect obese persons to be more concerned about their overall health state and hence be more aware of the presence of chronic diseases, as was the case with hypertension and hypercholesterolemia in our results. Longitudinal studies incorporating both quantitative and qualitative data collection may be warranted to verify our findings, elucidate underlying reasons and inform interventions to increase awareness of CVD risk factors in at-risk populations.

We also observed that self-reported concomitant presence of any of the three CVD risk factors and/or other comorbidities was associated with greater likelihood of awareness of the presence of all three CVD risk factors. In fact, further subgroup analyses of persons with all three CVD risk factors revealed a sharp decline in unawareness of each disease as the number of self-reported concomitant presence of the other two CVD risk factors increased (Additional file 1: Table S3). These findings are encouraging as they suggest that physicians are screening for the presence of all three conditions simultaneously. In addition, older individuals appeared to be more aware of having hypertension, possibly as a result of undergoing concomitant blood pressure checks during routine physician consultations; while being married was associated with greater awareness of hypercholesterolemia. The latter finding is not surprising as previous research has established that marriage is an important upstream determinant that influences exposure to cardiovascular risk factors, including awareness, social support and adherence to treatment [25]. Finally, individuals of Indian ethnicity and those who were unemployed were more likely to be aware of their diabetes status, which is heartening as the prevalence of diabetes is highest amongst Asian Indians [26] and unemployed individuals [27, 28], suggesting that diabetes awareness programs are effective in raising awareness amongst these at-risk individuals.

As suggested previously, we speculate that an individual’s unawareness of having a disease may be associated with poor control. While our results appear to uphold this theory, at least for hypertension and hypercholesterolemia, the converse appears to be true for persons who were unaware of having diabetes. We speculate that this could be because hypertension and hypercholesterolemia are generally asymptomatic and/or the symptoms are non-disease specific (e.g. headaches, nosebleeds) even when control is poor, while diabetes has well-recognized symptoms when glucose levels get dangerously high (e.g. polyuria, unexplained weight loss, coma). Therefore, patients with poor diabetes control are also the ones most likely to have sought medical advice for these symptoms. However, when we took a closer look at the data, we found that the proportion of individuals with HbA1c ≥ 9% were similar between both the aware and unaware groups (19.7% vs 17.6%). As such, the results do not appear to support our postulated theory; hence, future qualitative work to elicit possible reasons underpinning the better disease control observed in individuals unaware of having diabetes may be warranted. The data presented in this article nonetheless highlight the importance of improving the awareness of hypertension and hypercholesterolemia in order to improve control of the condition and reduce the risk of developing CVD.

Strengths of this study include the large population-based study design, with standardized assessment of the three conditions assessed in the study, as well as extensive quantitative information on the determinants and impact of the lack of awareness of each individual disease. Limitations include the cross-sectional design, which limits causal inferences; a lack of data on health and information-seeking behaviours, which may also contribute to awareness of an individual’s health state; and a lack of qualitative information on the factors underlying the lack of awareness of the three major CVD risk factors in our study participants. Moreover, our assessment of the three CVD risk factors does not necessarily follow accepted clinical protocols. For instance, hypertension was defined based on a single visit, which may have led to an overestimation of the prevalence of hypertensive individuals, with a consequent overestimation of the lack of awareness and its impact on disease control. However, these assessments are common practice in large-scale epidemiological studies due to time and resource constraints [16, 18, 29], and when we stratified our dataset according to whether participants were receiving treatment for each condition, those who were on treatment consistently showed higher levels of awareness and exhibited better disease control, save for diabetes, which is in line with our hypotheses (Additional file 1: Table S2). Finally, we excluded persons with CVD based on self-report. As such, there have been undiagnosed cases of CVD in our included population without CVD, which in turn may have led to an overestimation of unawareness.

## Conclusion

In conclusion, we showed that unawareness of diabetes, hypertension and hypercholesterolemia is highly prevalent in our multi-ethnic adult Singaporean population, with Malay individuals and smokers being most likely to be unaware of these conditions. In addition, unawareness was strongly associated with poor disease control in the case of hypertension and hypercholesterolemia. As such, public health education and screening campaigns should target individuals most likely to lack awareness of their health conditions in order to improve disease control and reduce the risk of CVD.

## Additional file

**Additional file 1: Table S1.** Baseline characteristics of patients by poor clinical control of diabetes, hypertension and hypercholesterolemia. **Table S2.** Awareness and control of diabetes, hypertension and hypercholesterolemia. **Table S3.** Association* between number of other self-reported CVD conditions with unawareness in patients with all three CVD conditions (*n*=1069).

## Data Availability

The datasets generated and/or analysed during the current study are not publicly available due to participant confidentiality issues but are available from the Singapore Eye Research Institute on reasonable request.

## Abbreviations

CVD: Cardiovascular disease
SERI: Singapore Eye Research Institute
HbA1c: Glycated hemoglobin
BMI: Body mass index
CI: Confidence interval
