# Supplementary material for: Prevalence, determinants and association of unawareness of diabetes, hypertension and hypercholesterolemia with poor disease control in a multi-ethnic Asian population without cardiovascular disease
Source: Popul Health Metr. 2019 Dec 5;17:17. doi: 10.1186/s12963-019-0197-5 (PMC6896313; doi:10.1186/s12963-019-0197-5)
Supplement: Supplementary file 1 — Additional file 1: Table S1. Baseline characteristics of patients by poor clinical control of diabetes, hypertension and hypercholesterolemia. Table S2. Awareness and control of diabetes, hypertension and hypercholesterolemia. Table S3. Association* between number of other self-reported CVD conditions with unawareness in patients with all three CVD conditions (n=1069). [file 12963_2019_197_MOESM1_ESM.docx]

| **Additional file 1: Table S1.** Baseline characteristics of patients by poor clinical control of diabetes, hypertension and hypercholesterolemia | | | | | | | | | |
| --- | --- | --- | --- | --- | --- | --- | --- | --- | --- |
|  | **Diabetes** | | | **Hypertension** | | | **Hypercholesterolemia** | | |
|  | **Mean (SD) / N (%)** | |  | **Mean (SD) / N (%)** | |  | **Mean (SD) / N (%)** | |  |
| **Variable** | **Good control (N: 1051)** | **Poor control^†^ (N: 1247)** | **P-value** | **Good control (N: 1211)** | **Poor control^†^ (N: 4173)** | **P-value** | **Good control (N: 1550)** | **Poor control^†^ (N: 1975)** | **P-value** |
| Age (years) | 62.3 (10.1) | 60.0 (9.50) | **<0.001** | 61.0 (9.67) | 61.6 (10.2) | 0.056 | 63.1 (9.20) | 58.3 (9.81) | **<0.001** |
| Body mass index (kg/m2) | 26.9 (4.92) | 27.0 (4.55) | 0.526 | 26.4 (4.73) | 25.9 (4.73) | **0.001** | 26.2 (4.45) | 25.4 (4.50) | **<0.001** |
| Number of self-reported non-CVD comorbid conditions* | 0.57 (0.73) | 0.50 (0.70) | **0.011** | 0.54 (0.73) | 0.46 (0.67) | **<0.001** | 0.61 (0.76) | 0.37 (0.60) | **<0.001** |
| Self-reported hypercholesterolemia | 586 (57.1) | 584 (48.1) | **<0.001** | 725 (60.9) | 1399 (34.4) | **<0.001** | 1426 (92.0) | 636 (32.2) | **<0.001** |
| Self-reported hypertension | 599 (57.0) | 626 (50.4) | **0.001** | 1082 (89.3) | 1983 (47.5) | **<0.001** | 1088 (70.3) | 520 (26.4) | **<0.001** |
| Self-reported diabetes | 657 (62.5) | 912 (73.1) | **<0.001** | 396 (32.8) | 940 (22.6) | **<0.001** | 702 (45.3) | 212 (10.8) | **<0.001** |
| Diabetes duration (years) | 5.54 (7.70) | 7.72 (8.97) | **<0.001** | N/A | | | N/A | | |
| **Gender** |  |  |  |  |  |  |  |  |  |
| Male | 498 (47.4) | 603 (48.4) | 0.642 | 560 (46.2) | 1974 (47.3) | 0.515 | 704 (45.4) | 858 (43.4) | 0.241 |
| Female | 553 (52.6) | 644 (51.6) |  | 651 (53.8) | 2199 (52.7) |  | 846 (54.6) | 1117 (56.6) |  |
| **Race** |  |  |  |  |  |  |  |  |  |
| Malay | 333 (31.7) | 513 (41.1) | **<0.001** | 255 (21.1) | 1696 (40.6) | **<0.001** | 302 (19.5) | 765 (38.7) | **<0.001** |
| Indian | 469 (44.6) | 501 (40.2) |  | 460 (38.0) | 1149 (27.5) |  | 628 (40.5) | 524 (26.5) |  |
| Chinese | 249 (23.7) | 233 (18.7) |  | 496 (41.0) | 1328 (31.8) |  | 620 (40.0) | 686 (34.7) |  |
| Income <S$1000 | 651 (63.5) | 752 (60.9) | 0.210 | 682 (57.6) | 2600 (63.4) | **<0.001** | 922 (60.9) | 1094 (56.0) | **0.004** |
| ≤ 6 years education | 736 (70.0) | 844 (67.7) | 0.238 | 757 (62.5) | 2878 (69.1) | **<0.001** | 973 (62.8) | 1245 (63.1) | 0.857 |
| Current smoker | 132 (12.6) | 178 (14.3) | 0.228 | 123 (10.2) | 590 (14.2) | **<0.001** | 140 (9.04) | 345 (17.5) | **<0.001** |
| Alcohol drinker | 77 (7.34) | 81 (6.51) | 0.432 | 103 (8.51) | 332 (7.97) | 0.550 | 112 (7.23) | 145 (7.35) | 0.890 |
| Lives alone | 60 (5.71) | 62 (4.98) | 0.437 | 63 (5.22) | 209 (5.02) | 0.773 | 69 (4.46) | 114 (5.78) | 0.079 |
| Works outdoor | 56 (5.33) | 80 (6.42) | 0.269 | 67 (5.53) | 240 (5.75) | 0.770 | 74 (4.77) | 129 (6.53) | **0.026** |
| **Occupation** |  |  |  |  |  |  |  |  |  |
| Professionals and office workers | 168 (16.0) | 175 (14.0) | 0.127 | 250 (20.6) | 556 (13.3) | **<0.001** | 267 (17.2) | 359 (18.2) | **<0.001** |
| Service workers, production workers or cleaners | 282 (26.8) | 378 (30.3) |  | 341 (28.2) | 1205 (28.9) |  | 393 (25.4) | 657 (33.3) |  |
| Homemakers, retirees, unemployed or others | 601 (57.2) | 693 (55.6) |  | 620 (51.2) | 2410 (57.8) |  | 890 (57.4) | 958 (48.5) |  |
| **Marital Status** |  |  |  |  |  |  |  |  |  |
| Never married | 44 (4.19) | 50 (4.02) | **0.015** | 62 (5.12) | 208 (4.99) | 0.737 | 61 (3.94) | 136 (6.89) | **<0.001** |
| Married | 787 (74.9) | 962 (77.3) |  | 919 (75.9) | 3115 (74.8) |  | 1189 (76.8) | 1485 (75.3) |  |
| Separated or divorced | 25 (2.38) | 50 (4.02) |  | 45 (3.72) | 151 (3.62) |  | 47 (3.03) | 90 (4.56) |  |
| Widowed | 195 (18.6) | 182 (14.6) |  | 185 (15.3) | 693 (16.6) |  | 252 (16.3) | 262 (13.3) |  |
| **Housing** |  |  |  |  |  |  |  |  |  |
| 5 room HDB and above | 285 (27.1) | 315 (25.3) | 0.318 | 394 (32.6) | 1090 (26.1) | **<0.001** | 530 (34.2) | 539 (27.3) | **<0.001** |
| 3-4 room HDB or less | 766 (72.9) | 931 (74.7) |  | 816 (67.4) | 3079 (73.9) |  | 1019 (65.8) | 1434 (72.7) |  |
| * Includes ocular (cataract, myopia, age-related macular degeneration, glaucoma, diabetic retinopathy, eye trauma) and systemic non-CVD (e.g. thyroid disease) conditions | | | | | | | | | |
| ^†^SBP ≥140mmHg or DBP ≥ 90mmHg for hypertension, HbA1c >7% for diabetes, and total cholesterol ≥6.2 mmol/L for hypercholesterolemia | | | | | | | | | |
| HDB=Housing Development Board | | | | | | | | | |

| **Additional file 1: Table S2.** Awareness and control of diabetes, hypertension and hypercholesterolemia | | | |
| --- | --- | --- | --- |
| Treatment (Yes; %) | Diabetes | Hypertension | Hypercholesterolemia |
| Unaware | 4.7 | 9.5 | 10.3 |
| Aware | 82.7 | 80.1 | 76.9 |
| Poor control (Yes; %) |  |  |  |
| Not on treatment | 46.7 | 95.0 | 100.0 |
| On treatment | 59.8 | 59.8 | 9.1 |

| **Additional file 1: Table S3.** Association* between number of other self-reported CVD conditions with unawareness in patients with all three CVD conditions (n=1069) | | | | | | |
| --- | --- | --- | --- | --- | --- | --- |
| Number of other self-reported CVD conditions | Diabetes | | Hypertension | | Hypercholesterolemia | |
|  | N (LOA %) | OR (95% CI) | N (LOA %) | OR (95% CI) | N (LOA %) | OR (95% CI) |
| 0 | 171 (56.1) | Ref | 142 (67.6) | Ref | 125 (76.8) | Ref |
| 1 | 244 (30.7) | 0.34 (0.22 to 0.52) | 294 (35.4) | 0.30 (0.19 to 0.47) | 310 (39.0) | 0.20 (0.12 to 0.33) |
| 2 | 654 (16.1) | 0.18 (0.12 to 0.27) | 633 (13.3) | 0.08 (0.05 to 0.13) | 634 (13.4) | 0.05 (0.03 to 0.09) |
| *P-trend^* |  | *<0.001* |  | *<0.001* |  | *<0.001* |
| *Adjusted for age, gender, ethnicity, body mass index, self-reported non-cardiovascular co-morbidity, income, education, smoking, alcohol use, occupation, marital status, housing  ^ Treating number of other self-reported CVD conditions as a continuous variable | | | | | | |
